# Gut Microbiome and Serum Metabolome Analyses Identify Unsaturated Fatty Acids and Butanoate Metabolism Induced by Gut Microbiota in Patients With Chronic Spontaneous Urticaria

**DOI:** 10.3389/fcimb.2020.00024

**Published:** 2020-02-21

**Authors:** Detong Wang, Shuping Guo, Hongxia He, Li Gong, Hongzhou Cui

**Affiliations:** ^1^Department of Dermatology, First Hospital of Shanxi Medical University, Taiyuan, China; ^2^The First Clinical Medical College of Shanxi Medical University, Taiyuan, China

**Keywords:** chronic spontaneous urticaria (CSU), pathogenesis, gut microbiota, serum metabolites, correlation analysis

## Abstract

Chronic urticaria (CU) is defined as the continuous or intermittent presence of urticaria for a period exceeding 6 weeks and sometimes occurring with angioedema. Between 66 and 93% of patients with CU have chronic spontaneous urticaria (CSU), the precise pathogenesis of which is largely unknown. The aim of this study was to determine the relationship between gut microbiota and serum metabolites and the possible pathogenesis underlying CSU. We collected feces and blood samples from CSU patients and healthy controls and the relationship between gut microbiota and serum metabolites was assessed using 16S rRNA gene sequencing and untargeted metabolomic analyses. The CSU group exhibited decreased alpha diversity of the microbial population compared to the control group. The abundance of unidentified *Enterobacteriaceae* was increased, while the abundance of *Bacteroides, Faecalibacterium, Bifidobacterium*, and unidentified *Ruminococcaceae* was significantly reduced in CSU patients. The serum metabolome analysis revealed altered levels of docosahexaenoic acid, arachidonic acid, glutamate, and succinic acid, suggesting changes in unsaturated fatty acids and the butanoate metabolism pathway. The combined serum metabolomics and gut microbiome datasets were correlated; specifically, docosahexaenoic acid, and arachidonic acid were positively correlated with *Bacteroides*. We speculate that alterations in gut microbes and metabolites may contribute to exacerbated inflammatory responses and dysregulated immune function with or without regulatory T cell dependence in the pathogenesis of CSU.

## Introduction

Chronic urticaria (CU) is defined by the continuous or intermittent presence of urticaria for a period exceeding 6 weeks and sometimes occurring with angioedema. CU can be classified into chronic inducible urticaria (CINDU) and chronic spontaneous urticaria (CSU) based on the triggering factor (Bernstein et al., 2014; Zuberbier et al., 2014b). Approximately 20% of individuals experience acute urticaria at least once during their lifetime, and 0.1% will develop chronic symptoms (Greaves, 2000; Zuberbier et al., 2014a). Between 66 and 93% of patients with CU have CSU. The main treatment option for CSU is to control symptoms, which significantly affect the quality of life patients (Fine and Bernstein, 2016).

The precise pathogenesis underlying CSU is largely unknown; however, CSU is considered to an immune-mediated inflammatory disease (Varghese et al., 2016). Gut microbiota has an important role in maintaining health through evolution and regulation of the immune system (Knight et al., 2017). Alterations in the gut microbiota has been shown to be associated with allergic diseases, including asthma (Arnold et al., 2011), food allergies (Ling et al., 2014), and atopic dermatitis (Hulshof et al., 2017). We previously conducted an epidemiologic investigation and found relevant evidence linking gut microbiota and CSU. Several studies have suggested that the changes in gut microbiota composition are linked to CU, including *Akkermansia muciniphila, Faecalibacterium prausnitzii*, the *Clostridium leptum* group, the Enterobacteriaceae family (Nabizadeh et al., 2017), and the *Lactobacillus, Bifidobacterium*, and *Bacteroides* genera (Rezazadeh et al., 2018); however, there is no specific mechanism by which to explore the dysbiosis of gut microbiota in the pathogenesis of CSU.

Combining the microbiome and metabolome may provide reliable and comprehensive information to elucidate the microbiologic-related pathogenesis underlying CSU and the metabolome can disclose the relevant metabolic pathways. Therefore, the aim of this study was to determine the relationship between gut microbiota and serum metabolites and the possible pathogenesis underlying CSU.

## Materials and Methods

### Study Subjects and Sample Collection

A total of 100 CSU patients and 100 healthy individuals (18–75 years of age) were enrolled in this study between June 2018 and June 2019. The diagnosis of CSU was established according to the American Academy of Allergy, Asthma, and Immunology criteria (Bernstein et al., 2014). The exclusion criteria were as follows: antibiotic use in the past month; abnormal levels of vitamin D and coagulation parameters; or any known disease (allergic rhinitis, asthma, eczema, obesity, and diabetes). A questionnaire was completed for each participant. We randomly selected 10 patients with CSU and 10 healthy individuals after matching for age and gender [mean [SD] age, 45.1 [4.94] years in each group; 5 [50%] males in each group] for further gut microbiome and serum metabolome analyses. All of the participants signed the informed consent. This study was approved by the Ethics Committee at the Shanxi Medical University. The principles of the 1964 Helsinki declaration and its later amendments were followed.

### Sample Collection, DNA Extraction, and 16S rRNA Sequencing

Approximately 2 g of fresh stool samples were collected from patients and healthy controls and frozen at −80°C until further studies. Bacterial DNA was extracted from stool samples using the CTAB/SDS method. Distinct region genes (16S rRNA/18S rRNA/ITS) were amplified specific primers (e.g., 16S-V4+V5, 515F: 5′-GTGCCAGCMGCCGCGGTAA-3′, 907R: 5′-CCGTCAATTCCTTTGAGTTT-3′) with barcodes before polymerase chain reaction (PCR) product mixing and purification were used. Sequencing libraries were generated and the library was sequenced on an Ion Plus Fragment Library Kit 48 rxns (Thermo Scientific, Philadelphia, Massachusetts, USA) after assessing quality on a Qubit@ 2.0 fluorometer (Thermo Scientific, Philadelphia, Massachusetts, USA). The last 400 bp/600 bp single-end reads were generated (Supplementary File 1).

### Bioinformatics Analysis of Microbiome Sequences

Sequence analysis was performed and a representative sequence for each operational taxonomic unit (OTUs) was screened for further annotation using Uparse software (Uparse v7.0.1001) (Edgar, 2013). Sequences with >97% similarity were assigned to the same OTU. Diversity analysis was performed, and the Silva Database (https://www.arb-silva.de/) was used based on the Mothur algorithm to annotate taxonomic information. To study the phylogenetic relationship of different OTUs, and the differences in the dominant species between samples (groups), multiple sequence alignment was performed using MUSCLE software (Version 3.8.31) (Edgar, 2004). Analysis of alpha and beta diversity was performed based on output normalized data. Tax4Fun functional prediction was achieved by the nearest neighbor method based on the minimum 16S rRNA sequence similarity (Supplementary File 1).

### Sample Collection, Metabolite Extraction, and UHPLC-MS/MS Analysis

Approximately 200 ul of serum samples were obtained from patients and healthy controls and stored at −80°C for further analysis. The filtrate was injected into the liquid chromatography-tandem mass spectrometry (LC-MS/MS) system after a series of treatments. LC-MS/MS analyses were performed using a Vanquish ultra-high-pressure liquid chromatograph (UHPLC) system (Thermo Fisher, Philadelphia, Massachusetts, USA) coupled with an Orbitrap Q Exactive HF-X mass spectrometer (Thermo Fisher, Philadelphia, Massachusetts, USA). The raw data files generated by UHPLC-MS/MS were processed using Compound Discoverer 3.0 (CD 3.0, Thermo Fisher, Philadelphia, Massachusetts, USA) to perform peak alignment, peak picking, and quantification for each metabolite. Then, peak intensities were normalized to the total spectral intensity. The normalized data were used to predict the molecular formula based on additive ions, molecular ion peaks, and fragment ions. The peaks were matched with the mzCloud (https://www.mzcloud.org/) and ChemSpider (http://www.chemspider.com/) databases to obtain the accurate qualitative and relative quantitative results. Finally, the functional and taxonomic of identified metabolites were annotated through KEGG (http://www.kegg.jp/) (Supplementary File 2).

### Data Analysis and Statistical Tests

Data were derived from the questionnaire using EpiData database double entry and the established database was analyzed by single factor analysis (χ^2^-test). The variables were analyzed by multi-factor logistic regression (*P* < 0.05) with SPSS23.0 statistical software. Alpha and beta diversity were calculated with QIIME (Version1.9.1) and displayed with R software (Version 2.15.3). Differential abundances of genera and metabolites were analyzed by parametric and non-parametric tests, including *t*-tests and Wilcoxon rank sum tests. Principal component analysis (PCA), fold change analysis, and partial least squares discriminant analysis (PLS-DA) were performed by MetaX software (Version 2.68). The metabolites with significant differences between 16S rRNA analysis and metabolomics analysis were analyzed based on the Pearson correlation coefficient. To further verify the authenticity of the correlation between bacteria and metabolites, a scatter plot was used. An adjusted *p*-value < 0.05 was considered statistically significant unless stated otherwise.

## Results

### Epidemiologic Investigation Indicated the Risk Factors for CSU

Two hundred participants completed the survey. There were 51 males and 49 females in the patient group, with a median age of 43.22 + 12.5 years. There were 48 males and 52 females in the healthy control group, with an average age of 39.04 + 10.6 years. No significant differences in age or gender existed between the two groups. Compared with the healthy controls, univariate analysis showed that place of residence, whether or not antibiotics were used during pregnancy, mode of production, feeding mode, dietary habits, whether, or not antibiotics were used in the past year, whether or not surgery was performed in the last 6 months, mental trauma and other stress factors, and whether or not alcohol consumption were significantly related to the onset of CSU (*P* < 0.05) (Supplementary Table 1). Multivariate logistic regression analysis showed that antibiotic use during pregnancy, cesarean section, a high-fat, low-carbohydrate diet, antibiotic use in the past year, and alcohol consumption were independent risk factors for CSU (Table 1).

**Table 1:** Multivariate logistic regression analysis of clinical data of two groups of patients.

**Variable**	**OR-value (95%CI)**	***P*-value**
Whether antibiotics are used in pregnancy	2.730 (1.353 ~ 5.511)	0.005
Mode of production	2.534 (1.246 ~ 5.152)	0.010
Eating habits	1.933 (1.271 ~ 2.938)	0.002
Whether antibiotics are used in the last year	4.475 (2.260 ~ 8.859)	0.000
Whether or not drinking	1.530 (0.994 ~ 2.355)	0.050

### Diversity and Richness of the Gut Microbiota in CSU Decreased Significantly

**Figure 1:**
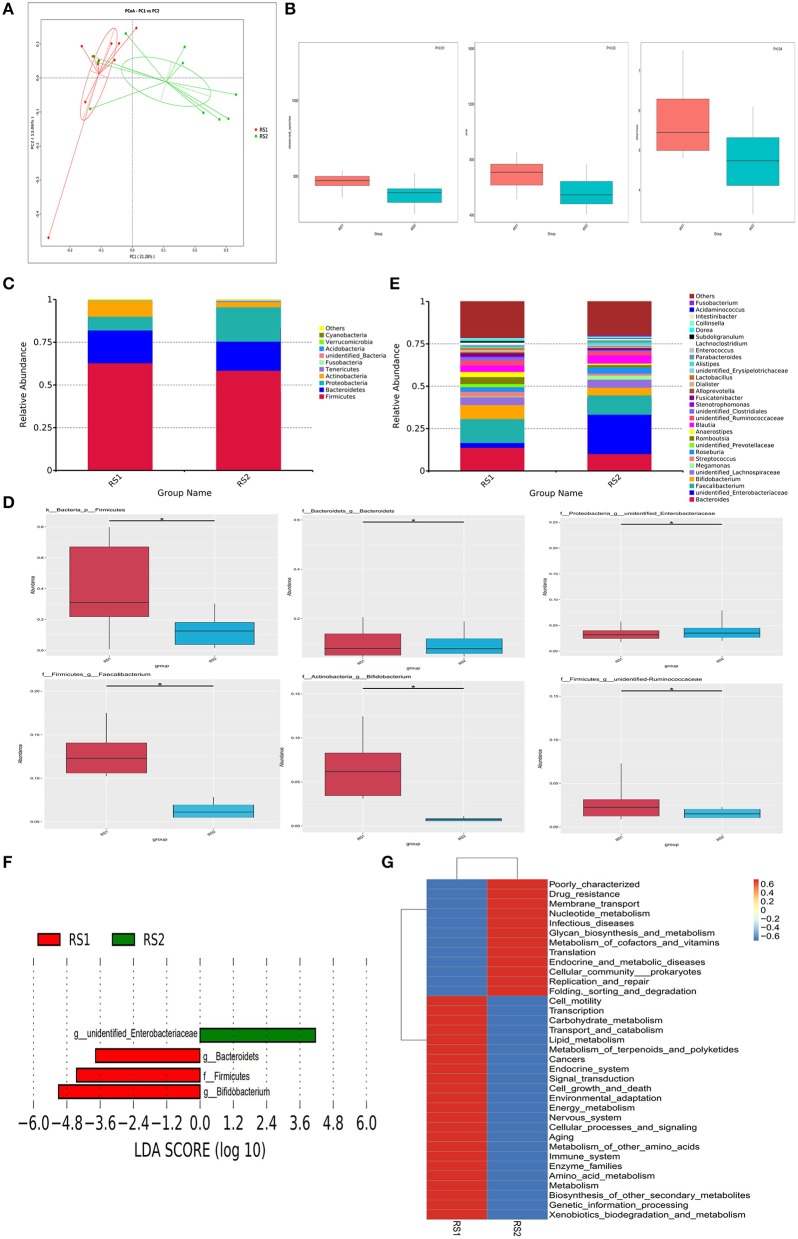
Microbiome multivariate analysis. **(A)** Principal Component Analysis (PCoA) based on unweighted UniFrac distances between gut bacterial communities of CSU patients and healthy controls. **(B)** Alpha-diversity indexes for each sample group, showing the adjusted *p*-value computed using Wilcoxon rank sum test. **(C)** Bacterial composition and abundance in phylum level. Each bar represents the top ten bacterial species ranked by the relative abundance in CSU patients and healthy controls. **(D)** The relative abundance of significantly altered bacterial taxa including phylum, genus levels in the CSU group compared to that in the control group (*P < 0.05). **(E)** Taxonomic distributions of bacteria at the genus level (top 30) between CSU patients and healthy controls. **(F)** Lefse analysis was performed on the bacterial taxa relative abundance values between the two groups. Bacterial with LDA score >2.0 and *P* < 0.05 were considered to be significantly discriminant. **(G)** The effect of the altered gut microbiota on predicted functional metabolic pathways.

After quality control, an average of 76,183 valid data were obtained, 1,983 OTUs were identified, then the OTU sequence and Silva Database were annotated. A total of 1,010 (50.93%) OTUs were annotated to the genera level. Participants were divided into the two groups in the Principal Co-ordinates Analysis (PCoA) (Figure 1A). Alpha diversity was applied to determine the ecologic diversity, including observed species, ACE, and Shannon. There were significant differences (*P* = 0.01) in the observed species measured between CSU patients and healthy controls (Figure 1B). Both the ACE and Shannon indices reflect the reduced community richness, evenness, and species diversity in CSU patients, which showed a statistically significant difference (Figure 1B).

### Gut Microbiota Showed Differences in Phyla and Genera Levels in CSU

As shown in Figure 1C, the two groups were mainly composed of *Firmicutes, Bacteroidetes, Proteobacteria*, and *Actinobacteria*. *Firmicutes* is the most prominent gut bacterial community, accounting for 63 and 59% of CSU patients and healthy controls, respectively. At the phylum level, the relative abundance of *Firmicutes* in CSU patients was markedly decreased compared to healthy controls (Figure 1D). At the genus level, members of the *Bacteroides, Faecalibacterium, Bifidobacterium, Lactobacillus*, and unidentified *Ruminococcaceae* were relatively lower and unidentified *Enterobacteriaceae* were relatively increased compared to healthy controls (Figure 1E). And unidentified *Enterobacteriaceae* were increased, while *Bacteroides, Faecalibacterium, Bifidobacterium*, and unidentified *Ruminococcaceae* were decreased in CSU patients (Figure 1D).

### Gut Bacterial Taxa Were Significantly Altered in CSU Patients

LEfSe analysis showed that the CSU patients were characterized by a higher abundance of unidentified *Enterobacteriaceae*, whereas the healthy controls primarily showed higher enrichment with *Firmicutes, Bacteroides* and *Bifidobacteriales* (LDA score >2.0 with *P* < 0.05) (Figure 1F). Tax4Fun functional prediction mainly indicated that modifications of the gut microbiota may lead to changes in related metabolic pathways, such as nucleotide metabolism (Figure 1G).

### Cluster Analysis Identified Differential Abundant Metabolites in CSU Patients

**Figure 2:**
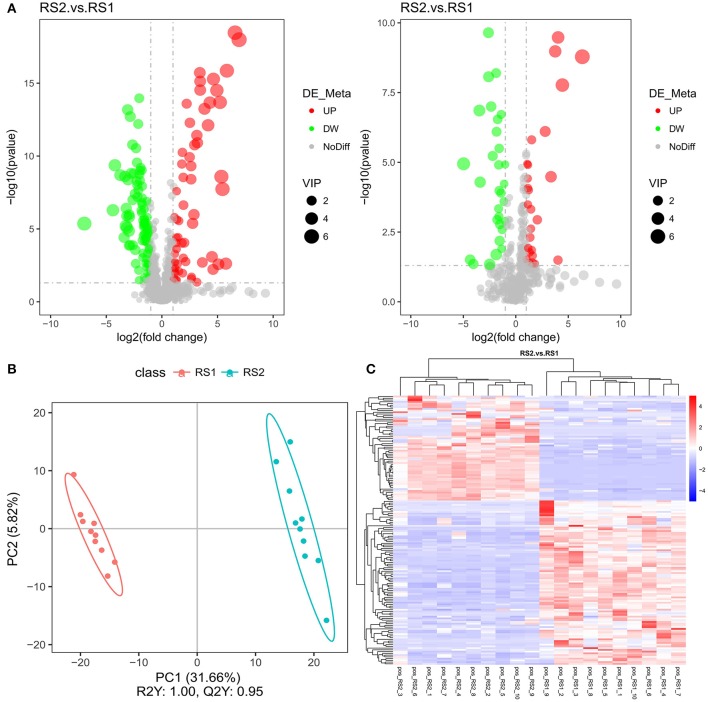
Metabolic profile multivariate analysis. **(A)** Volcano plot of the identified metabolites in positive ion mode (left) and in negative ion mode (right). **(B)** Plot of OPLS-DA scores of the control (red) and CSU (blue) groups. **(C)** The heatmap showed the abundance of metabolites in different clusters from each sample.

LC-MS/MS analyses separated the identified metabolite profiles into positive and negative ion modes (Want et al., 2010; Dunn et al., 2011). The number of differential metabolites in the two groups was 154 in the positive ion mode, of which 60 were significantly up-regulated, and 56 were in the negative ion mode, of which 26 were significantly up-regulated (Figure 2A). These metabolites included amino acids (glutamic acid and succinic acid), fatty acids (docosahexaenoic acid and arachidonic acid), carbohydrates (sucrose), and purines (Supplementary File 3). The PLS-DA scores showed an obvious separation trend in serum metabolic profiles between the CSU and control groups (Figure 2B). The heatmap analysis revealed a remarkable difference between the composition of CSU serum metabolites and the healthy controls (Figure 2C).

### Correlation Analysis Identified Unsaturated Fatty Acid and Butanoate Metabolism

**Figure 3:**
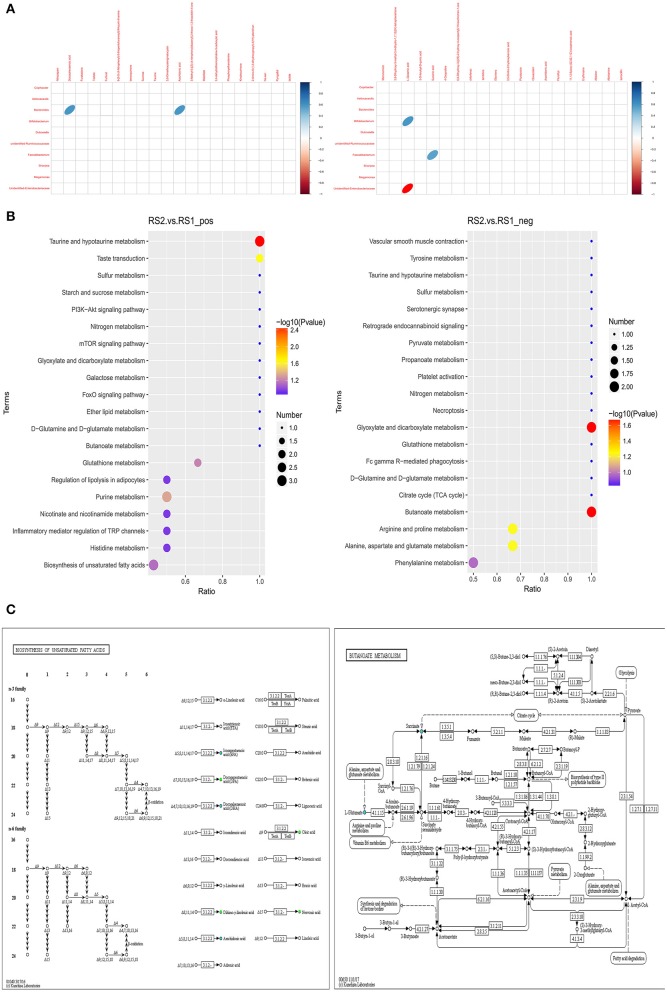
Correlation analysis of differential gut microbiota and metabolites. **(A)** Heatmap of correlation between microbial genus abundances and serum metabolites in positive ion mode (left) and in negative ion mode (right). Only significant correlations (*p* ≤ 0.05) are colored. Positive correlations are indicated in blue and negative correlations in red. **(B)** Metabolites were annotated into different metabolic pathways between the positive ion mode (left) and in negative ion mode (right) by KEGG enriched bubble chart based on KEGG. **(C)** Metabolic pathway map in CSU patients. Green solid circles are marked as annotated metabolites, and blue circles are marked as down-regulated differential metabolites.

Spearman correlation analysis showed that docosahexaenoic acid and arachidonic acid had a positive correlation with *Bacteroides*. L-glutamic acid had a negative correlation with unidentified *Enterobacteriaceae* and a positive correlation with *Bifidobacterium*. Succinic acid showed a positive correlation with *Faecalibacterium* (Figure 3A). According to the KEGG-enriched bubble chart, we demonstrated biosynthesis of unsaturated fatty acids and butanoate metabolism in the positive and negative ion modes, respectively. The former has significant difference between CSU patients and healthy controls (Figure 3B). Interestingly, the KEGG pathway map results indicated that docosahexaenoic acid and arachidonic acid belongs to the biosynthesis of unsaturated fatty acid metabolism, while L-glutamic acid and succinic acid were products of butanoate metabolism (Figure 3C).

## Discussion

We demonstrated that antibiotic use during pregnancy, cesarean section, high-fat, low-carbohydrate diets, antibiotic use in the past year, and alcohol consumption are independent risk factors for CSU. Recent evidence revealed that a high-fat diet in mice altered the intestinal microbiota composition; specifically, the number of *Bifidobacterium* were significantly reduced (Zhou et al., 2019). Several studies have shown that the administration of antibiotics can lead to disorders of intestinal flora and decreased microbial diversity (Angelucci et al., 2019; Sun et al., 2019). Therefore, through epidemiologic investigation, we found an indirect relationship between intestinal microbial and the incidence of CSU.

We further analyzed the composition and diversity of microbes by Illumina-based 16S rRNA gene sequencing. Both the ACE and Shannon indices reflected a reduction in the bacterial diversity and richness in CSU patients, which is highly consistent with other allergic diseases (Bisgaard et al., 2011). At the phylum level, when compared with healthy controls, there was a relative reduced abundance of *Bacteroidetes* and *Firmicutes* in CSU patients, which is partly in agreement with the airway allergies in young children (Chiu et al., 2019). At the genus level, members of the *Bacteroides, Faecalibacterium, Bifidobacterium, Lactobacillus*, and unidentified *Ruminococcaceae* were relatively lower and members of unidentified *Enterobacteriaceae* were relatively increased compared to healthy controls, which is partly consistent with previous studies about CU or allergic diseases (Sjogren et al., 2009; Johansson et al., 2011; Candela et al., 2012; Nabizadeh et al., 2017; Rezazadeh et al., 2018; Chiu et al., 2019). For example, Nabizadeh et al. (2017) and Rezazadeh et al. (2018) reported a depletion in the relative amounts of *Faecalibacterium prausnitzii, Clostridia, Akkermansia muciniphila, Bifidobacterium*, and *Lactobacillus* in CU patients. The mean of the relative amounts of *Enterobacteriaceae* was increased. In the current study, we did not detect *Akkermansia muciniphila* in the top 30 bacterial species at the genus level.

The genus *Bacteroides* is defined as a gram-negative, rod-shaped, obligate anaerobic that plays an important role in maintaining immune homeostasis and regulating immunity and has an association with unsaturated fatty acids (Lee et al., 2003). Recent research has examined that reduced maternal n-3 polyunsaturated fatty acid exposure resulted in a significant decrease of *Epsilonproteobacteria, Bacteroides*, and *Akkermansia* and increased relative abundance of *Clostridia* (Robertson et al., 2018). Similar results have been reported that unsaturated fatty acids have a stimulating effect on the growth of *Bacteroides* (Mirjafari Tafti et al., 2019). Our findings showed a significant reduction in *Bacteroides* in patients with CSU and a positive correlation between docosahexaenoic acid and arachidonic acid. Furthermore, these two metabolites were down-regulated in the unsaturated fatty acid biosynthesis pathway. Unsaturated fatty acids have anti-inflammatory properties and suppresses inflammatory responses (Calder and Grimble, 2002; Morrison and Preston, 2016). Therefore, we concluded that the reduction in unsaturated fatty acids exacerbated the inflammatory responses to promote the development of CSU.

The metabolome also confirmed that there were differences in butanoate metabolism between CSU patients and healthy controls. Butyrate is involved in the maintenance of intestinal epithelial cells and plays a significant role in regulating intestinal immune tolerance to antigens (Chang et al., 2014). Recent evidence revealed that the abundance of butyrate was decreased in children with asthma and was negatively correlated with serum IgE levels. This suggests that the dysregulation of the intestinal epithelial barrier due to reduced butyrate secretion may be related to allergen uptake and immunological responses in allergic asthma (Chiu et al., 2019). We detected a decrease in the serum levels of glutamate and succinic acid in CSU patients which both belong to butanoate metabolism. More importantly, a reduction in *Bifidobacterium* and *Faecalibacterium*, which had a positive correlation with glutamate and succinic acid, respectively. The increased in unidentified *Enterobacteriaceae* had a negative correlation with glutamate. The results indicated that lower butyrate levels due to dysregulation of the gut microbiota may play an important role in the pathogenesis of CSU.

*Ruminococcus* is a genus of strictly anaerobic, Gram-positive, non-motile cocci, and is one of the most predominant organisms in the colon from the phylum Firmicutes (Hold et al., 2002; La Reau and Suen, 2018). Arshi et al. reported that a reduction in the population of *Ruminococcus* in the gut induces inflammatory responses by Toll-like receptors and subsequently participates in the development of eczema in infancy (Arshi et al., 2014). In our study, the abundance of unidentified *Ruminococcaceae* was significantly lower in CSU patients, which is consistent with previous studies of allergic diseases (West et al., 2015; Simonyte Sjödin et al., 2016). In addition, several previous studies demonstrated that the number and function of regulatory T (Treg) cells was reduced in CU patients (Chen et al., 2008; Arshi et al., 2014). In summary, we speculate that depletion of certain bacteria, such as *Ruminococcus*, may modulate the immune function of Treg cells by inducing Toll-like receptors contributing to CSU.

Our study revealed profound changes in the composition of gut microbes-metabolites and the corresponding relationship, including *Bacteroides* associated with docosahexaenoic acid and arachidonic acid, *Bifidobacterium, Faecalibacterium*, and unidentified *Enterobacteriaceae* associated with glutamate and succinic acid, and the decreased abundance of *Ruminococcus*. Taken together, we speculate that the changes may contribute to exacerbated inflammatory responses and dysregulated immune function with or without Treg cell dependence in the pathogenesis of CSU. The major limitation of this study was the relatively small population of participants. More research is needed to understand the mechanism underlying these associations.

## Data Availability Statement

All datasets generated for this study are included in the article/Supplementary Material.

## Ethics Statement

The studies involving human participants were reviewed and approved by Ethics Committee at the Shanxi Medical University. The patients/participants provided their written informed consent to participate in this study.

## Author Contributions

HH and LG collected samples and made clinical records. HC, DW, and LG performed the experiments. HC conceived the study and will oversee all aspects of the project. DW and SG wrote the first draft of this manuscript. All authors read, approved the final manuscript, and made an intellectual contribution to this study.

### Conflict of Interest

The authors declare that the research was conducted in the absence of any commercial or financial relationships that could be construed as a potential conflict of interest.
