# Supplementary material for: Gut Microbiome and Serum Metabolome Analyses Identify Unsaturated Fatty Acids and Butanoate Metabolism Induced by Gut Microbiota in Patients With Chronic Spontaneous Urticaria
Source: Front Cell Infect Microbiol. 2020 Feb 21;10:24. doi: 10.3389/fcimb.2020.00024 (PMC7047433; doi:10.3389/fcimb.2020.00024)
Supplement: Supplementary file 1 [file Table_1.doc]

Supplementary Table 1

Table 1 Risk factors for single factor analysis of clinical data in two groups of patients

| Risk factors | | Number of patients | Number of healthy persons | χ^2^-value | *P*-value |
| --- | --- | --- | --- | --- | --- |
| Place of residence | city | 74 | 55 | 7.883 0.005 | |
|  | Rural | 26 | 45 |  |  |
| Whether antibiotics are used in pregnancy | no | 45 | 74 | 17.450 0.000 | |
|  | yes | 55 | 26 |  |  |
| Mode of production | Natural childbirth | 51 | 73 | 10.272 0.001 | |
|  | Caesarean birth | 49 | 27 |  |  |
| Feeding mode | Pure breast milk | 29 | 36 | 8.789 0.032 | |
|  | Mixed feeding (breast milk > formula milk powder) | 29 | 23 |  |  |
|  | Mixed feeding (breast milk < formula milk powder) | 22 | 33 |  |  |
|  | Pure formula milk powder | 20 | 8 |  |  |
| Eating habits | A low-fat, high-carbohydrate diet | 9 | 32 | 20.465 0.000 | |
|  | Medium-fat medium-carbohydrate diet | 43 | 43 |  |  |
|  | A high-fat, low-carbohydrate diet | 41 | 20 |  |  |
|  | Others | 7 | 5 |  |  |
| Whether antibiotics are used in the last year | no | 31 | 66 | 24.522 0.000 | |
|  | yes | 69 | 34 |  |  |
| Stress factors such as surgery, trauma and so on in the past six months | no | 61 | 81 | 9.713 0.002 | |
|  | yes | 39 | 19 |  |  |
| Whether or not drinking | No drinking | 33 | 59 | 14.754 0.002 | |
|  | Less than 100g/day | 51 | 28 |  |  |
|  | 100-250g/day | 12 | 11 |  |  |
|  | More than 250 g/day | 4 | 2 |  |  |

Note: Low-fat high-carbohydrate diet for meat <50 grams or fat supply calories <1/6, carbohydrates> 2/3; Medium-fat carbohydrate diet is 1/6 <fat supply calories <1/3, 1/3<Carbohydrates<2/3;High-fat and low-Carbohydrate diet for fats provide calories >1/3 and carbohydrates<1/3;Others include the ratio of food, vegetables and meat is 1:2:3 or high fat , high protein and low carbohydrate.
