# Supplementary material for: Gut Microbiome and Serum Metabolome Analyses Identify Unsaturated Fatty Acids and Butanoate Metabolism Induced by Gut Microbiota in Patients With Chronic Spontaneous Urticaria
Source: Front Cell Infect Microbiol. 2020 Feb 21;10:24. doi: 10.3389/fcimb.2020.00024 (PMC7047433; doi:10.3389/fcimb.2020.00024)
Supplement: Supplementary file 2 [file Data_Sheet_1.doc]

Supplementary File 1

Illumina-based 16S rDNA gene sequencing methods

Sequencing

1. Extraction of genome DNA

Total genome DNA from samples was extracted using CTAB/SDS method. DNA concentration and purity was monitored on 1% agarose gels. According to the concentration, DNA was diluted to 1 ng/µL using sterile water.

2. Amplicon Generation

16S rRNA/18S rRNA/ITS genes of distinct regions (16S V4 / 16S V3 / 16S

V3-V4 / 16S V4-V5, 18S V4 / 18S V9, ITS1 / ITS2, Arc V4, et. al) were amplified

used specific primer (e.g. 16S V4: 515F-806R, 18S V4: 528F-706R, 18S V9:

1380F-1510R, et. al) with the barcode. All PCR reactions were carried out in 30 µL

reactions with 15 µL of Phusion® High-Fidelity PCR Master Mix (New England

Biolabs); 0.2 µM of forward and reverse primers, and about 10 ng template DNA.

Thermal cycling consisted of initial denaturation at 98℃for 1 min, followed by 30

cycles of denaturation at 98℃for 10 s, annealing at 50℃for 30 s, and elongation at 72℃ for 30s. Finally 72℃for 5 min.

3. PCR Products Mixing and Purification

Mix same volume of 1×loading buffer (contained SYB green) with PCR products and operate electrophoresis on 2% agarose gel for detection. PCR products was mixed in equidensity ratios. Then, mixture PCR products was purified with GeneJETTM Gel Extraction Kit (Thermo Scientific).

4. Library preparation and sequencing

Sequencing libraries were generated using Ion Plus Fragment Library Kit 48 rxns

(Thermo Scientific) following manufacturer's recommendations. The library quality

was assessed on the Qubit@ 2.0 Fluorometer (Thermo Scientific). At last, the library

was sequenced on an Ion S5TM XL platform and 400 bp/600 bp single-end reads were generated.

Data analysis

1. Single-end reads quality control

1.1 Data split

Single-end reads was assigned to samples based on their unique barcode and truncated by cutting off the barcode and primer sequence.

1.2 Data Filtration

Quality filtering on the raw reads were performed under specific filtering conditions to obtain the high-quality clean reads according to the Cutadapt [1] (V1.9.1, http://cutadapt.readthedocs.io/en/stable/) quality controlled process.

1.3 Chimera removal

The reads were compared with the reference database (Silva database, https://www.arb-silva.de/) [2] using UCHIME algorithm (UCHIME Algorithm, http://www.drive5.com/usearch/manual/uchime_algo.html) [3] to detect chimera sequences, and then the chimera sequences were removed [4] . Then the Clean Reads

finally obtained.

2. OTU cluster and Species annotation

2.1 OTU Production

Sequences analysis were performed by Uparse software (Uparse v7.0.1001， http://drive5.com/uparse/) [5] . Sequences with ≥97% similarity were assigned to the

same OTUs. Representative sequence for each OTU was screened for further annotation.

2.2 Species annotation

For each representative sequence, the Silva Database (https://www.arb-silva.de/) [2] was used based on Mothur algorithm to annotate taxonomic information.

2.3 Phylogenetic relationship Construction

In order to study phylogenetic relationship of different OTUs, and the difference

of the dominant species in different samples (groups), multiple sequence alignment

were conducted using the MUSCLE software (Version 3.8.31，http://www.drive5.com/muscle/) [6].

2.4 Data Normalization

OTUs abundance information were normalized using a standard of sequence

number corresponding to the sample with the least sequences. Subsequent analysis of

alpha diversity and beta diversity were all performed basing on this output normalized

data.

3. Alpha Diversity

Alpha diversity is applied in analyzing complexity of species diversity for a

sample through 6 indices, including Observed-species, Chao1, Shannon, Simpson,

ACE, Good-coverage. All this indices in our samples were calculated with QIIME

(Version1.7.0) and displayed with R software (Version 2.15.3). Two indices were

selected to identify Community richness:

Chao - the Chao1 estimator (http://www.mothur.org/wiki/Chao);

ACE - the ACE estimator (http://www.mothur.org/wiki/Ace);

Two indices were used to identify Community diversity:

Shannon - the Shannon index (http://www.mothur.org/wiki/Shannon);

Simpson - the Simpson index (http://www.mothur.org/wiki/Simpson);

One indice to characterized Sequencing depth:

Coverage - the Good’s coverage (http://www.mothur.org/wiki/Coverage)

4. Beta Diversity

Beta diversity analysis was used to evaluate differences of samples in species

complexity, Beta diversity on both weighted and unweighted unifrac were calculated

by QIIME software (Version 1.7.0). Cluster analysis was preceded by principal component analysis (PCA), which was applied to reduce the dimension of the original variables using the FactoMineR package and ggplot2 package in R software (Version 2.15.3). Principal Coordinate Analysis (PCoA) was performed to get principal coordinates and visualize from complex, multidimensional data. A distance matrix of weighted or unweighted unifrac among samples obtained before was transformed to a new set of orthogonal axes, by which the maximum variation factor is demonstrated by first principal coordinate, and the second maximum one by the second principal coordinate, and so on. PCoA analysis was displayed by WGCNA package, stat packages and ggplot2 package in R software (Version 2.15.3). Unweighted Pair-group Method with Arithmetic Means (UPGMA) Clustering was performed as a type of hierarchical clustering method to interpret the distance matrix using average linkage and was conducted by QIIME software (Version 1.7.0).

5. Funnction Prediction

Tax4Fun functional prediction was achieved by the nearest neighbor method

based on the minimum 16S rRNA sequence similarity by extracting the KEGG database prokaryotic whole genome 16S rRNA gene sequence and aligning it to the SILVA SSU Ref NR database using BLASTN algorithm (BLAST Bitscore >1500) to

establish a correlation matrix, and map the prokaryotic whole genome functional information of the KEGG database annotated by UProC and PAUDA to the SILVA database to implement the SILVA database function annotation. The sequenced samples were clustered out of the OTU using the SILVA database sequence as a reference sequence to obtain functional annotation information.

Reference

[1] Martin M. Cutadapt removes adapter sequences from high-throughput sequencing

reads[J]. Embnet Journal, 2011, 17(1).

[2] Quast C, Pruesse E, et al. The SILVA ribosomal RNA gene database project:

improved data processing and web-based tools[J]. Nucl. Acids Res. (2013):

D590-D596.

[3] Edgar, Robert C., et al. UCHIME improves sensitivity and speed of chimera

detection[J]. Bioinformatics 27.16 (2011): 2194-2200.

[4] Haas, Brian J., et al. Chimeric 16S rRNA sequence formation and detection in

Sanger and 454-pyrosequenced PCR amplicons[J]. Genome research 21.3 (2011):

494-504.

[5] Edgar, Robert C. UPARSE: highly accurate OTU sequences from microbial

amplicon reads[J]. Nature methods 10.10 (2013): 996-998.

[6] Edgar R C. MUSCLE: multiple sequence alignment with high accuracy and high

throughput[J]. Nucleic acids research32.5 (2004): 1792-1797.

.
