# Supplementary material for: Gut Microbiome and Serum Metabolome Analyses Identify Unsaturated Fatty Acids and Butanoate Metabolism Induced by Gut Microbiota in Patients With Chronic Spontaneous Urticaria
Source: Front Cell Infect Microbiol. 2020 Feb 21;10:24. doi: 10.3389/fcimb.2020.00024 (PMC7047433; doi:10.3389/fcimb.2020.00024)
Supplement: Supplementary file 3 [file Data_Sheet_2.doc]

Supplementary File 2

Untargeted Metabolomics - Materials and Methods

1. prechilled methanol (400 μL) were individually grounded with liquid nitrogen and the homogenate was resuspended with prechilled 80% methanol and 0.1% formic acid by well vortexing. The samples were incubated on ice for 5 min and then were centrifuged at 15000 rpm, 4°C for 5 min. A some of supernatant was diluted to final concentration containing 60% methanol by LC-MS grade water. The samples were subsequently transferred to a fresh Eppendorf tube with 0.22 μm filter and then were centrifuged at 15000 g, 4°C for 10 min. Finally, the filtrate was injected into the LC-MS/MS system analysis.
2. UHPLC-MS/MS Analysis LC-MS/MS analyses were performed using a Vanquish UHPLC system (Thermo Fisher) coupled with an Orbitrap Q Exactive HF-X mass spectrometer (Thermo Fisher). Samples were injected onto an Hyperil Gold column (100×2.1 mm, 1.9μm) using a 16-min linear gradient at a flow rate of 0.2 mL/min. The eluents for the positive polarity mode were eluent A (0.1% FA in Water) and eluent B (Methanol). The eluents for the negative polarity mode were eluent A (5 mM ammonium acetate, pH 9.0) and eluent B (Methanol). The solvent gradient was set as follows: 2% B, 1.5 min; 2-100% B, 12.0 min; 100% B, 14.0 min；100-2% B, 14.1 min；2% B, 16 min. Q Exactive HF-X mass spectrometer was operated in positive/negative polarity mode with spray voltage of 3.2 kV, capillary temperature of 320°C, sheath gas flow rate of 35 arb and aux gas flow rate of 10 arb.

3. Data analysis The raw data files generated by UHPLC-MS/MS were processed using the Compound Discoverer 3.0 (CD 3.0, Thermo Fisher) to perform peak alignment, peak picking, and quantitation for each metabolite. The main parameters were set as follows: retention time tolerance, 0.2 minutes; actual mass tolerance, 5ppm; signal intensity tolerance, 30%; signal/noise ratio, 3; and minimum intensity ,100000. After that, peak intensities were normalized to the total spectral intensity. The normalized data was used to predict the molecular formula based on additive ions, molecular ion peaks and fragment ions. And then peaks were matched with the mzCloud (https://www.mzcloud.org/) and ChemSpider (http://www.chemspider.com/) database to obtained the accurate qualitative and relative quantitative results.
